# Case Report: Molecular diagnostics and clinical courses of two adult spinal pilocytic astrocytoma long-term survivors with GTF2I::BRAF fusion

**DOI:** 10.3389/fonc.2026.1670639

**Published:** 2026-03-04

**Authors:** Lorenzo Argao, Pinar E. Zerk, Hsiang-Chih Lu, Zied Abdullaev, Martha Quezado, Michelle L. Cassidy, Bennett Mclver, Anna Choi, Marissa Panzer, Renee Tweneboah-Koduah, Lily Polskin, Marta Penas-Prado, Paul Park, Nathan Clarke, Kenneth Aldape, Jacob Mandel, Byram H. Ozer

**Affiliations:** 1 Neuro-Oncology Branch, National Cancer Institute, Bethesda, MD, United States; 2 Laboratory of Pathology, National Cancer Institute, Bethesda, MD, United States; 3 Department of Pathology and Immunology, Baylor College of Medicine, Houston, TX, United States; 4 Department of Neurosurgery, University of Michigan Medical School, Ann Arbor, MI, United States; 5 Department of Neurology, University of Michigan Medical School, Ann Arbor, MI, United States; 6 Department of Neurology, Dan L. Duncan Comprehensive Cancer Center, Baylor College of Medicine, Houston, TX, United States; 7 Department of Medical Oncology, Winship Cancer Institute, Emory University Hospital, Atlanta, GA, United States

**Keywords:** BRAF fusion, case report, methylation profiling, pilocytic astrocytoma, spinal tumor

## Abstract

**Introduction:**

Pilocytic astrocytomas are driven by BRAF and mitogen-activated protein kinase (MAPK) alterations, typically KIAA1549::BRAF fusions. A rare GTF2I::BRAF fusion has been described, but little is known about these cases.

**Case report:**

Here, we report two cases with GTF2I::BRAF fusions. Case 1 is a 36-year-old man initially diagnosed with myxopapillary ependymoma at the conus medullaris with three recurrences over 23 years requiring two surgeries, three rounds of radiation therapy, and one round of lapatinib/temozolomide. A distant disease focus in T3/T4 was sampled and tested with modern diagnostic techniques revealing a pilocytic astrocytoma on histology and methylation profiling. The patient has subsequently had stable clinical and radiographic findings. Case 2 is another 36-year-old man initially diagnosed with meningitis and later neurosarcoid who underwent biopsy after 12 years when his spinal leptomeningeal disease continued to progress and an intraventricular non-enhancing nodule emerged as a separate focus. Sampling of the leptomeningeal disease led to a diagnosis of pilocytic astrocytoma by histology and a divergent methylation profile. The patient has remained neurologically stable under radiographic surveillance without any intervention.

**Results:**

Radiographic, histological, and molecular data are presented for both cases and compared against the only other reported GTF2I::BRAF CNS case, as well as canonical versions of pilocytic astrocytoma.

**Conclusion:**

To our knowledge, this is only the second case series highlighting a unique GTF2I::BRAF fusion and the first to describe it in adults in a spinal location. The manuscript contributes documentation of a rare fusion and tumor presentation to guide clinicians and potential research avenues.

## Introduction

Low-grade gliomas encompass a wide range of diagnoses, genetics, and clinical outcomes. By far, the most common is pilocytic astrocytoma (PA), which is a benign, Grade 1 glioma that accounts for approximately 20.1% of glioma diagnoses in the pediatric population and 1.2% of all CNS tumors. The average annual age-adjusted incidence is 0.37 cases per 100,000, and they are commonly diagnosed in the first two decades of life (1). Survivorship well into adulthood is common as many PAs are curable by surgery alone.

PA is driven by mitogen-activated protein kinase (MAPK) pathway alteration, a signaling cascade involved in proliferation, cell-cycle arrest, terminal differentiation, and apoptosis (2). Most PAs are located in the cerebellum, and nearly half of all PA cases have the KIAA1549::BRAF fusion as its driver (3), the result of a tandem duplication event that typically fuses KIAA1549 at a breakpoint in exon 16 with BRAF at exon 9. This leads to loss of the N-terminal regulatory domain and constitutive activity of the BRAF kinase domain independent of RAS signaling (4). When stratified by age, KIAA1549::BRAF fusion is present in up to 79% of those diagnosed in the first decade of life and decreases steadily every decade to <10% when diagnosed in patients over 40 (5). In the absence of KIAA1549::BRAF fusion, other MAPK pathway drivers of PA include mutations in NF1 (negative regulator of Ras) (6), FGFR [receptor tyrosine kinase (RTK) activator of MAPK pathway] (7), and other BRAF alterations, most commonly BRAF^V600E^ mutation. These alternative drivers are loosely associated with clinical and/or diagnostic features. For example, NF1 mutations are more common in optic pathway PAs and can have a more aggressive clinical course while FGFR1 hotspot mutations, fusions, and internal tandem duplications are more commonly found in brainstem PAs and in adults (8). Similarly, BRAF^V600E^ mutations in PAs may have a predilection for more diffuse, though not necessarily more aggressive, growth patterns (9).

We describe here two cases of spinal PA in adults with a rare fusion mutation, GTF2I::BRAF, previously only described once in the literature to our knowledge (10). We seek to expand on this single reported case to look at trends in diagnosis, radiological presentation, and clinical outcomes.

## Case presentation

### Case 1

**Figure 1:**
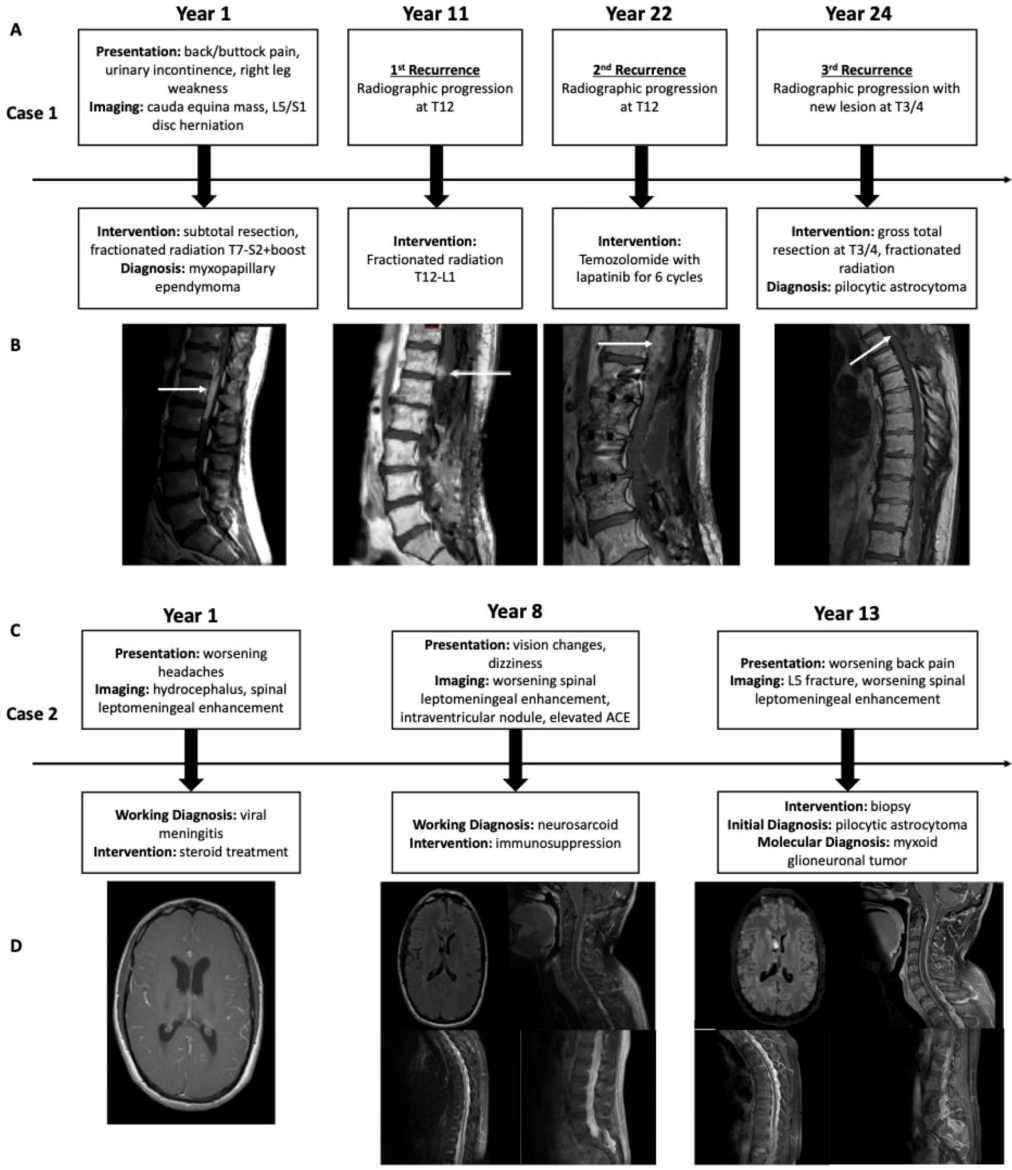
Case 1 timeline of clinical events and treatments **(A)** with associated radiographic progression **(B)**. Case 2 timeline of clinical events and treatments **(C)** with associated radiographic progression **(D)**.

A 36-year-old man presented to his local physician with a 2-month history of right buttock/perineum pain, right leg weakness, and difficulty with urination. Lumbar spine magnetic resonance imaging (MRI) showed a mass at the conus medullaris and cauda equina along with an L5-S1 disc herniation. The patient underwent a subtotal resection of the T11-L2 mass (Year 1), which was diagnosed as a Grade 2 myxopapillary ependymoma (diagnosis made in 2001 in the pre-molecular era, original tissue not available for re-review, and no CSF staging work-up available). He received 45 Gy of radiation to T7-S2 with boost to 54 Gy to L1-S2. Though needing several laminectomies to address other areas of stenosis and spinal cord compression, his tumor remained stable for 10 years (Year 11, first recurrence) when surveillance imaging showed a worsening tumor focus superior to the initial lesion at T12. He was treated empirically with an additional 50.4 Gy of fractionated radiation to T12-L1. He again had two additional spine surgeries for re-exploration of previously operated areas, but his tumor remained stable until 2022 when the T12 focus showed progression (Year 22, second recurrence) and for which he started six cycles of temozolomide with lapatinib as indicated for recurrent ependymomas (11). Genetic counseling was seen as part of the standard work-up. However, with no strong family history and with familial inheritance patterns unlikely for ependymoma, no strong indication for testing was identified and the patient declined further work-up. While this recurrent lesion remained stable after treatment, a new focus at T3/T4 developed gradually over the ensuing 2 years (Year 24, third recurrence). This was believed to be a recurrence and not a radiation-induced mass given the relative distance from the high-dose radiation fields, though treatment planning maps were not available to confirm. He elected to undergo a surgical resection of this tumor followed by a course of 50.4 Gy fractionated radiation (**Figure 1A**), which was administered due to the relatively fast growth rate of the new mass, previous response to radiation, and relative safety as the new lesion was likely distant from high-dose fields in previous radiation plans. At his last follow-up (Year 25), he has remained radiographically stable (**Figure 1B**) but over the prior decades has suffered from a steady decline in his mobility due to worsening lower extremity weakness and is needing some support, such as canes and walking sticks, for walking longer distances.

**Figure 2:**
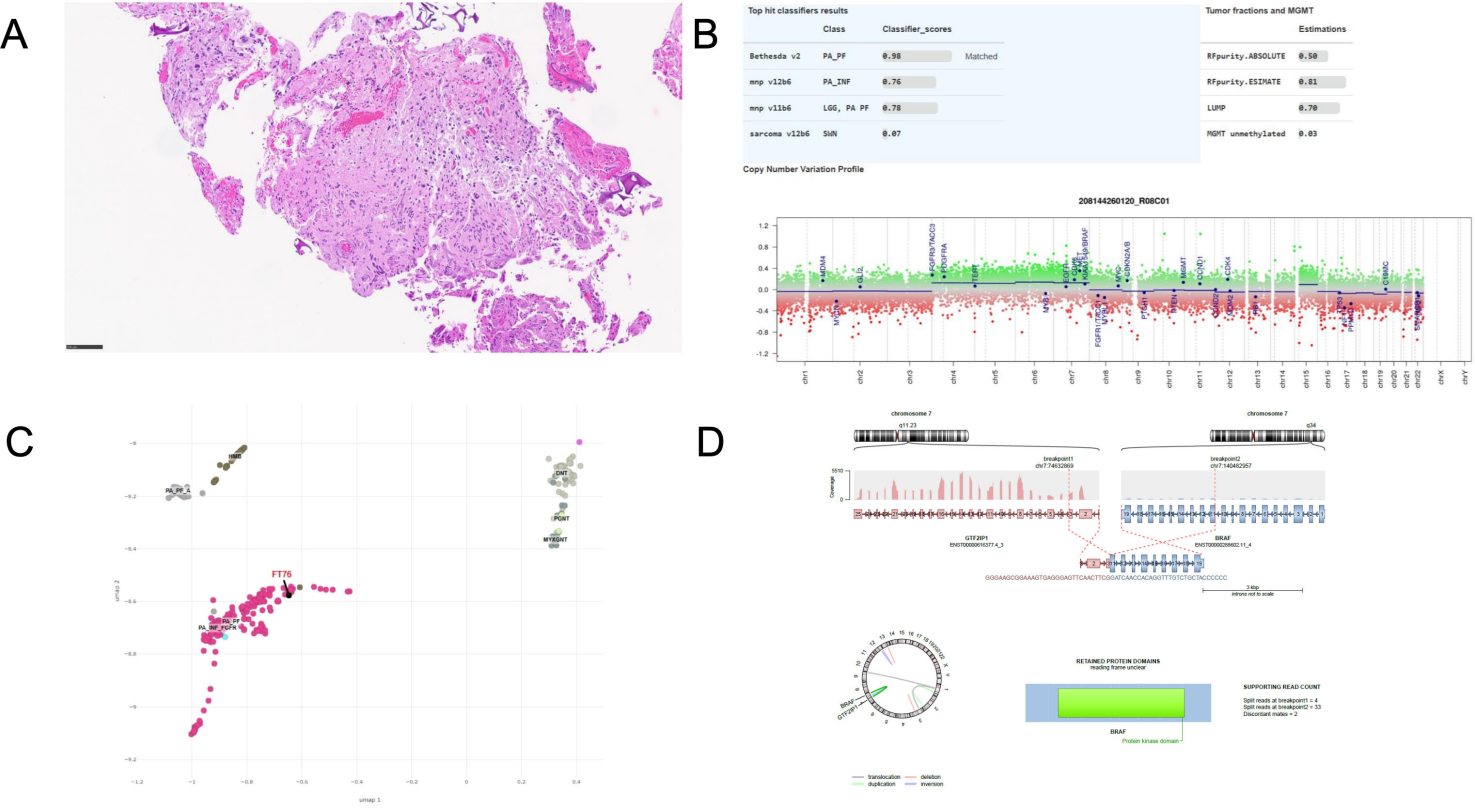
Diagnostic work-up from Case 1. **(A)** Histological micrograph showing glial neoplasm of low to moderate cellularity, neoplastic cells showing varying proportions of piloid and oligodendrocyte-like cells, as well as round to elongated nuclei with scattered multinucleated cells (horseshoe-shaped nulcei) consistent with pilocytic astrocytoma. **(B)** Methylation profile showing high score match to pilocytic astrocytoma, posterior fossa (PA-PF) and flat copy number variant results. **(C)** UMAP localizing to PA-PF and proximity to other low-grade neoplasms. **(D)** Fusion map derived from RNAseq showing breakpoints and proposed protein product consisting primarily of intact BRAF kinase domain.

With his original tumor tissue no longer available, his T3/4 tumor was used for diagnostic evaluation. Histologically, hematoxylin and eosin (H&E) staining showed low to moderate cellularity, neoplastic cells composed of a mixture of piloid and oligodendrocyte-like morphologies, and scattered multinucleated cells with horseshoe-shaped nuclei that are characteristic of PA (**Figure 2A**). Methylation profiling by Bethesda classifier version 2.0 matched the tumor to pilocytic astrocytoma, posterior fossa (PA_PF) with very high confidence (score 0.98), with a flat copy number profile (**Figure 2B**), and appropriately embedded in the UMAP (**Figure 2C**). The DKFZ classifiers confirmed the diagnosis, albeit with below-threshold scores (score 0.76). No mutations were detected on NGS, and RNA sequencing (RNAseq) revealed a fusion between GTF2I at exon 3 and BRAF at exon 11. The reading-frame prediction is uncertain but ostensibly produces a protein product characterized by a fully intact BRAF kinase domain with a small, N-terminal portion of the GTF2I protein product Transcription Factor II-I (TFII-I) replacing the N-terminal regulatory domain of BRAF (**Figure 2D**).

### Case 2

A 36-year-old man presented with a history of worsening headaches (Year 1). Head imaging showed evidence of hydrocephalus and leptomeningeal enhancement, raising concerns for viral meningitis. He was treated with steroids, which had little effect, improving instead on acetazolamide and NSAID therapy. Seven years later (Year 8), he presented with new vision changes and dizziness. MRI of the CNS axis revealed diffuse leptomeningeal enhancement throughout the spinal cord, including the subarachnoid space of the lumbar spinal canal, as well as nodular enhancement along the nerves of the cauda equina. Brain MRI also revealed a non-enhancing nodule within the right lateral ventricle that did not appear related. CSF studies detected elevated ACE levels, which raised suspicion for neurosarcoid but with no biopsy performed to confirm. He subsequently started immunosuppressants, as well as acetazolamide and meloxicam for symptom management, but his imaging never responded. Five years later (Year 13), he developed worsening back pain and lumbar spine imaging showed a spinal fracture at L5, worsening diffuse leptomeningeal enhancement throughout the spine, and minimal change in the intraventricular lesion. Given the continued diagnostic uncertainty, he underwent a biopsy of the thickening leptomeningeal enhancement at L5/S1 (**Figure 1C**). Histologically, the diagnosis was consistent with PA, and the patient was neurologically asymptomatic other than back discomfort. The treating team discussed options with the patient that included radiation, targeted inhibitor therapy, and clinical trials. However, the risk/reward of radiation to include the lesion and the leptomeningeal enhancement was felt to not be favorable in the absence of symptoms, which equally applied to the available clinical trial. At that time, targeted inhibitor therapies were not effective against BRAF activating fusions. The patient therefore opted for surveillance until clinical or radiological progression, and a second opinion at a separate institution supported this decision. At the last follow-up (Year 18), the patient has remained both clinically and radiographically stable (**Figure 1D**) and has undergone no further treatment.

**Figure 3:**
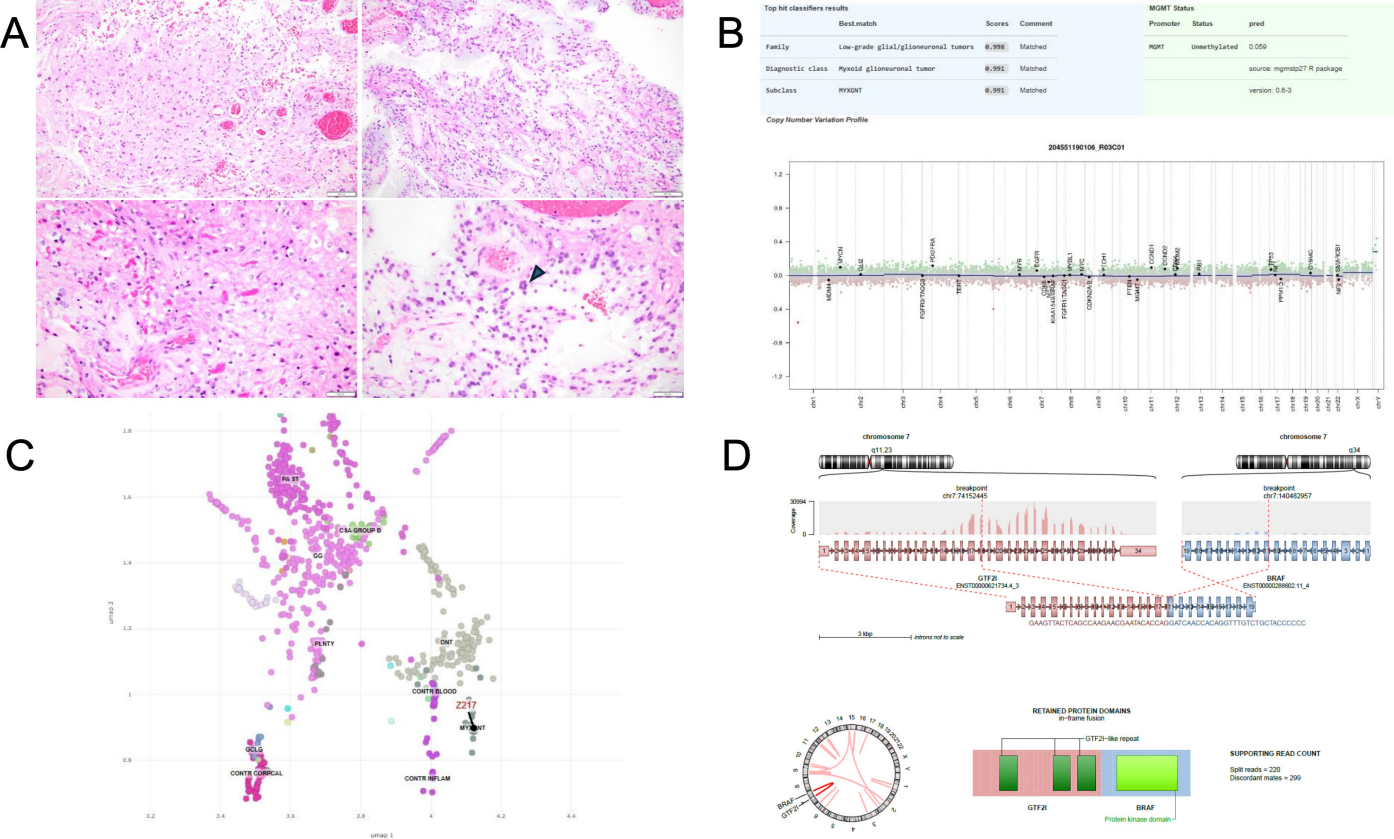
Diagnostic work-up from Case 2. **(A)** Histological micrographs showing glial neoplasm with hyalinized vessels and dense fibrillary areas (top left) alternating with loose microcystic areas (top right), focally abundant Rosenthal fibers (bottom left), and multinucleated cells (pennies on a plate, bottom right) consistent with pilocytic astrocytoma. **(B)** Methylation profile showing high score match to myxoid glioneuronal tumor and flat copy number variant results. **(C)** UMAP localizing to MGT and proximity to other low-grade neoplasms. **(D)** Fusion map derived from RNAseq showing breakpoints and proposed protein product consisting of DNA-binding domains of GTF2I and intact BRAF kinase domain.

Histologically, the biopsy from the L5/S1 leptomeningeal enhancing region showed a glial neoplasm with hyalinized vessels and alternating dense fibrillary and loose microcystic areas with abundant Rosenthal fibers and occasional multinucleated cells, all consistent with PA (**Figure 3A**). Subsequent methylation profiling unexpectedly resulted as myxoid glioneuronal tumor (MGT) with high confidence on the Bethesda Classifier version 2.0 (score 1), which was supported on the DKFZ classifiers with low confidence (score 0.6). Interestingly, a separate block from the same tumor was consistent with DNET on the DKFZ classifiers, albeit again with low confidence (score 0.61) (**Figure 3B**). The sample also embedded with MGT samples on the UMAP (**Figure 3C**). Next-generation sequencing showed an NF2 variant of unknown significance (VUS) and a MUTYH heterozygous germline variant, but no BRAF or MAPK driver mutation typical of PA or any PDGFRA alteration typical of MGT. RNAseq detected a fusion between GTF2I at exon 18 and BRAF at exon 11, whose in-frame fusion protein product generates an intact BRAF protein kinase domain without the N-terminal regulatory domain, as well as sufficient TFII-I protein to retain three of its repeat domains (**Figure 3D**), its N-terminal leucine zipper, and the nuclear localization domain (12). A referral was made to genetic counseling, and genetic testing revealed a heterozygous MUTYH mutation and an NF2 missense VUS. The genetic counselor determined that the heterozygous MUTYH mutation conferred a marginally increased risk of colorectal cancer and no increased risk of extraintestinal tumors including in the CNS.

## Discussion and patient perspective

Low-grade gliomas commonly have BRAF fusions as their driver, with the majority composed of PAs in cerebellar locations and with pediatric onset. We describe here two unusual cases of spinal PAs with GTF2I::BRAF fusions diagnosed in adulthood and compare them against canonical KIAA1549::BRAF fusion PAs and the only other reported case of GTF2I::BRAF fusion-positive PA (**Table 1**). Because methylation profiling and UMAP in Case 2 was consistent with MGT despite classical histological findings of PA, we also include canonical MGT as a further basis of comparison.

**Table 1:** Comparison of demographic, clinical, and radiological features of Case 1 and Case 2 compared to the PA case from the literature and canonical presentations of PA and MGT.

Age at presentation	Adult (36)	Adult (36)	Adolescent (17)	Child > Adolescent	Child > Adolescent
Sex	Male	Male	Male	Male = Female	Male > Female
Tumor location	Spine (lesional)	Spine (LM) + brain (IV)	Posterior fossa	Posterior fossa	Intraventricular, septum pellucidum
Initial diagnosis	Myxopapillary ependymoma	Pilocytic astrocytoma	Pilocytic astrocytoma	N/A	N/A
Methylation classifier	PA	MYXGNT	Not done	PA	MYXGNT
Fusion exons	GTF2I: 3 BRAF: 11	GTF2I: 18 BRAF: 11	GTF2I: 19 BRAF: 10	KIAA1549: 16 BRAF: 9	N/A
Copy number plot	Balanced diploid	Balanced diploid	Not reported	Balanced diploid	Balanced diploid, rare gain of 12q (19)
Fusion product	TFII-I:? BRAF: KD ? Frame?	TFII-I: R1-R3, BR, LZ, NLS BRAF: KD In-frame	TFII-I: R1-R3, BR, LZ, NLS BRAF: KD In-frame	UPF0606:? BRAF: KD In-frame	N/A
MRI presentation	Enhancing LM with distant nodules	Diffusely enhancing LM with non-enhancing intraventricular nodule and hydrocephalus	Cystic mass with irregular peripheral/nodular enhancement; separate pituitary enhancing mass	Cystic mass with mural nodular enhancement is classical	Non-enhancing, intraventricular mass along septum pellucidum, hydrocephalus
Mutations	None	Germline MUTYH, non-specific NF2	N/A	None	PDGFRA K385L/I
Clinical course	Slowly and multiply progressive along spine	Slowly progressive	No progression after resection (36 months)	Progression uncommon after resection and adulthood	Progression uncommon but may occur within 1–5 years
Recurrence	Yes (10 years)	No	No	Uncommon	Uncommon
Survival	25+ years	18+ years	3+ years	Decades	Decades

BR, bromodomain; LM, leptomeninges; LZ, leucine-zipper; MYXGNT, myxoid glioneuronal tumor; NLS, nuclear localization signal; PA, pilocytic astrocytoma; R1–3, repeat domains 1–3.

Case 1 differs from PAs not just because of its non-canonical BRAF fusion, but also because of older age of onset and its diffuse presentation as multinodular spinal disease with multiple recurrences and undergoing numerous treatment courses. Typically, PAs arise from the posterior fossa with a very classical cyst and mural nodular enhancement appearance on MRI, and both recurrence and treatment needs are uncommon after initial resection. It is difficult to determine whether a multiply recurrent disease course is a distinguishing feature: all pediatric-onset PAs have the possibility of recurring and becoming treatment refractory, a patient cohort highly represented in the tovorafenib BRAF-inhibitor studies designed for pediatric recurrent low-grade gliomas (13). Some also suggest that adult-onset PAs may be more aggressive (14), but this hypothesis stems from the pre-molecular era that neither accounted for mutations nor guaranteed disease homogeneity, and biological understanding, systemic therapies, and surgical techniques have also since improved (15). Furthermore, because the original diagnosis was myxopapillary ependymoma WHO Grade 2, rendered with the 2000 WHO diagnostic criteria in a pre-molecular era, this very likely influenced treatment decisions. It cannot be entirely ruled out that the PA diagnosis at T3/4 is a separate and new tumor focus apart from the lumbosacral spine tumor, and there is no original tumor tissue available for testing, but we consider two separate tumors an unlikely scenario and that the diagnosis of myxopapillary ependymoma was likely PA all along. The literature case documenting a 17-year-old male patient with a posterior fossa lesion (10) differs from Case 1 both in the composition of the GTF2I::BRAF fusion and in the clinical course that radiographically and clinically resembles canonical PAs.

Case 2 had a much more diffuse and less lesional appearance compared to Case 1, but otherwise shared characteristic histological features, a predominance in the spine, and a slowly developing clinical course. Though the histology was classical for PA, the methylation profiling unexpectedly matched MGT with a high score with a corresponding match on UMAP. Although methylation profiling is a very accurate diagnostic tool and often performs better than histology on diagnostics, in this case, the classic histomorphology findings of PA combined with the molecular findings provided a more accurate diagnosis and expose limitations to methylation profiling in its current iteration, especially in low-cellularity and leptomeningeal samples. For one, MGTs are confined solely to the septum pellucidum, with only very rare reports of slowly progressive leptomeningeal disease (16). Furthermore, MGTs are almost definitionally associated with PDGFRA mutation (17, 18), specifically at the hotspot of K385L/I (19). The presence of a novel BRAF fusion without any PDGFRA alteration in Case 2 would be a substantial departure from the norm for MGT. Similarly, there were none of the characteristic NGS or RNAseq findings showing FGFR alterations (mutation, fusion, and amplification) that are associated with DNET. Finally, it is well-known in the methylation profiling literature that the diagnostic accuracy to distinguish between pediatric low-grade glial and glioneuronal neoplasms is not as robust compared to other CNS tumors. This is due to diverse cell types, both normal and abnormal, influencing the algorithm, and differences in methylation algorithm interpretations. In gliomas where there is a characteristic mutation (such as BRAF fusion), the recommendation is to favor the diagnostic interpretation most consistent with the mutation rather than the epigenetic profile (20). Presumably future refinement will improve these algorithms. An additional point of consideration in the differential diagnosis of Case 2 is diffuse leptomeningeal glioneuronal tumor (DLGNT). DLGNT shares many properties with PA, including frequent BRAF fusions as oncogenic drivers. In this case, the immunohistochemical diagnosis strongly favored PA. Despite DLGNT having a very distinctive cluster on methylation profiling, in a case like this where there is discrepancy between methylation and immunohistochemical/molecular diagnostics, DLGNT belongs on the differential. Oligodendrogliomas can also occasionally share features with spinal PA, but the combination of radiological appearance, biological activity, and immunohistochemical findings did not prompt any follow-up testing for 1p/19q co-deletion.

In KIAA1549::BRAF fusions, oncogenesis is derived from loss of the BRAF regulatory N-terminal domain and constitutive C-terminal kinase activity rather than a gain of function from KIAA1549 (4). *KIAA1549* encodes a protein product belonging to the UPF0606 family, but there is minimal understanding of its usual function (21). While the Case 1 fusion, with minimal GTF2I contribution, is likely kinase domain-driven similar to canonical BRAF fusions, it is unclear if the Case 2 GTF2I::BRAF fusion has separate GTF2I-related oncogenic driving functions beyond constitutive BRAF kinase activation. Past evaluations of a similar protein product demonstrated clear downstream ERK phosphorylation and activation (10) similar to canonical BRAF fusion products. However, the GTF2I protein product TFII-I has well-elucidated DNA-binding and transcriptional functions, and much of this domain is intact in the literature-reported case and Case 2. The TFII-I domain retains its leucine zipper and basic region involved in DNA binding, as well as its Y248 residue that binds phosphorylated ERK as a necessary precondition for nuclear translocation to initiate transcription (22). It is therefore conceivable that the GTF2I fusion protein domains may work in concert with MAPK pathway hyperactivation (23), but any additional contribution beyond this in the setting of fusions is unknown (12).

The incidental discovery of a MUTYH mutation in Case 2 warrants further discussion. Though not an established germline driver of PA, it is always appropriate to refer patients to genetic counseling for clinical and germline assessments when known cancer predisposing mutations are found. This is not only to assess the patient’s overall cancer risk, especially in diseases such as PA where long-term survival is expected, but also to evaluate and look for new genetic predisposition trends that may alert providers to screen for CNS tumors in the future. Indeed, several case reports have posited a possible relationship between germline MUTYH mutation and pediatric-type gliomas (24, 25). Even if a clear cancer predisposition mutation is not found in tumor tissue, there is an ongoing debate about whether there might be benefit to obtaining germline testing on all rare CNS cancer patients. The etiology and oncogenic mechanisms are poorly understood, and looking for trends in genetic alterations (SNPs, gene mutation VUS, etc.) may help to establish these trends, but will never be known if not routinely assessed and tested.

Irrespective of the highlighted molecular and radiographic similarities and differences, these low-grade gliomas with GTF2I::BRAF appear to follow indolent disease courses where long-term neurological symptom management is the main comorbidity, but clinical follow-up is still ongoing. Nonetheless, both patients continue with surveillance monitoring without further intervention and lead normal lives with good quality of life. Though symptoms of neurological dysfunction persist, with gradually worsening gait and balance in the first case and headaches in the second, they are well-managed and provide only modest interference to daily life. Fortunately, with the advent of Type II pan-RAF kinase inhibitor therapy (e.g., tovorafenib), there are now well-tolerated therapies that are effective against BRAF activating fusions and may provide clinical and radiographic relief, either as a salvage treatment or as a means of delaying traditional radiation and systemic therapies (13).

## Conclusion

PAs are primarily driven by KIAA1549::BRAF fusions. We describe here the clinical course and associated diagnostics from low-grade gliomas driven by novel GTF2I::BRAF fusions. It remains unclear if all iterations of this fusion are biologically equivalent or whether they are specifically associated with demographic and other clinical features, and we hope that reporting these cases contributes to the expanding understanding of CNS tumors and their unique molecular drivers.

## Data Availability

Data presented is de-identified clinical and molecular data from two patients as part of a case report. De-identified details of the data can be furnished upon request. Requests to access these datasets should be directed to Byram.Hirsch.Ozer@emory,.edu.
